# "Good outcome after drowning with prolonged resuscitation and secondary pediatric acute respiratory distress syndrome requiring extra-corporeal-membrane-oxygenation – A special case in comparison to the single-center local 10-year experience in pediatric drowning”

**DOI:** 10.3389/fped.2026.1841402

**Published:** 2026-06-10

**Authors:** Kai Fiedler, Chris Mohrmann, Lisa Meyer, Christiaan Peter Sentner, Axel Heep

**Affiliations:** 1 Pediatric Intensive Care Unit, University Hospital, Department of Neonatology/Pediatric Intensive Care/Pediatric Cardiology/Pediatric Pulmonology, Carl von Ossietzky Universität Oldenburg, School VI - School of Medicine and Health Sciences, Oldenburg, Germany; 2 Carl von Ossietzky Universität Oldenburg, School VI - School of Medicine and Health Sciences, Oldenburg, Germany

**Keywords:** neurologic outcome, pediatric ARDS, pediatric drowning, pediatric ECMO, pediatric emergency, resuscitation

## Abstract

Drowning is a leading cause of morbidity and mortality in childhood accidents. We report the case of a 13 year old boy who after prolonged resuscitation developed severe secondary ARDS requiring ECMO. Despite the presence of multiple risk factors for poor outcome prognosis he recovered well and was discharged in an age-appropiate neurologic condition. We compare this case to our local 10-year-experience in childhood drowning and similar reports in the literature to identify the special circumstances leading to this unexpected outcome. This analysis reveals no significant differences or specific parameters at the initiation of intensive care treatment that would explain the discrepancy between the presence of multiple risk factors and the positive clinical outcome. Thus, decision-making in pediatric drowning accidents remains uncertain.

## Introduction

Worldwide, drowning is a common cause of morbidity and mortality in children (1). There are several risk factors for poor outcome like prolonged submersion- and resuscitation-time (2, 3). Development of pediatric acute respiratory disstress syndrome (pARDS) and multi-organ failure during the stay in the pediatric intensive care unit (PICU) may contribute to the described risk (4, 5). Here we report the case of a 13 year old boy who developed severe pARDS after prolonged resuscitation in a drowning event requiring extra-corporeal membrane oxygenation (ECMO). He survived reaching pre-accidental neurologic status at hospital discharge despite having several poor prognostic risk factors. We compared this case to our preexisting 10-year experience with childhood drowning to identify the special circumstances leading to this unexpected outcome.

## Materials and methods

### Retrospective data of childhood drowning cases

We included all patients 0–18 years of age suffering from a drowning event with subsequent stay in the PICU at the University Children`s Hospital in Oldenburg, Germany, from January 2013 until August 2023, identified by international classification of diseases code T75.1 (ICD 10). Epidemiological characteristics and vital signs were collected from preclinical records, at the time of arrival at the emergency department (ED), on arrival in the PICU and during the first 24 h in the PICU. There were no substantial changes in treatment protocol over the whole study period. Outcome data were collected at patient discharge from the PICU. All data were extracted from the hospital patient data management system (Cerner KIS medico®). Analysis included basic parameters such as cause of accident and influencing factors (submersion time, water temperature, body temperature at presentation and development during the ICU stay).

Severity of illness within the first hour of admission to the PICU was retrospectively calculated by Pediatric Index of Mortality (PIM-3) (6). Neurological status at admission and before discharge from the PICU was retrospectively calculated by Functional Status Scale (FSS) based on clinical data within patients’ records (7).

For the case report, patient record and laboratory data were analyzed from admission until discharge from the PICU. All available data from the electronic patient data management system and the individual patient record were used.

### Statistical analysis

All clinical data were extracted from the electronic patient data management system Medico (Cerner KIS medico®) to charts in MS Excel® (Microsoft Corporation). According to low patient counts mainly descriptive statistical analyses were performed. With parameters showing a wide range of values we report median values, all other numbers are shown as average values where applicable.

If applicable, analysis of statistical significance with Fisher Exact-Test was done by online-calculator at socscistatistics.com (https://www.socscistatistics.com/tests/fisher/calculator).

### Ethics and consent

Patient data and conduct of the case during intensive care is provided with informed consent of the parents. Data collection of the local pediatric drowning cases was approved by the local medical ethics board (No. 2023-185).

## Case-report

We report a 13-year old male patient (weight: 50 kg, length: 150 cm) with preexisting chronic epilepsy due to Lennox-Gastaux-Syndrome who experienced a drowning accident in a public indoor swimming pool during a swimming lesson. Submersion time was estimated to have been 5 min. Resuscitation was begun immediately upon retrieval from the water. The emergency medical service (EMS) team arrived about 10 min later and continued resuscitation. The preclinical electrocardiography showed pulseless electrical activity (PEA). After intraosseous access was established, repetitive doses of epinephrine were administered. Initial ventilation by laryngeal mask was replaced by endotracheal intubation due to oral bleeding. Successive mechanical ventilation was uncomplicated. After 20 min of ongoing resuscitation PEA persisted, so the patient was transferred to the hospital with ongoing mechanical resuscitation (Lucas®-Device, Stryker).

On arrival at the ED pulmonary ventilation had deteriorated with significant amounts of bloody endobronchial secretions resulting in lowered SpO_2_ and hypercarbia (pCO_2_ 76 mm Hg). Glasgow coma scale (GCS) was 3 with equally wide pupils after repeated epinephrine doses. Core body temperature was 35,5 °C. During initial assessment the patient had return of spontaneous circulation (ROSC) after a total resuscitation time of 95 min and echocardiography showed adequate biventricular cardiac function without pericardial effusion. An arterial and central venous line was inserted. Laboratory parameters are shown in Table 1.

**Table 1:** Laboratory parameters at admission.

Parameter	Value
pH	6,67
Arterial pCO_2_	76 mmHg
Arterial pO_2_	129 mmHg
HCO_3−_	5 mmol/L
Base excess	−25 mmol/L
Lactate	15 mmol/L
Glucose	464 mg/dL
Potassium	4,5 mmol/L
Hemoglobin	15,7 g/dL
Quick	26%
aPTT	>160s
Fibrinogen	<60 mg/dL
D-dimer	>30 mg/L

**Figure 1:**
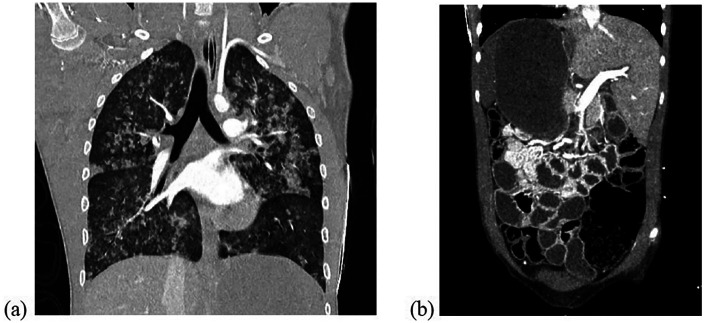
CT chest with bilateral infiltration **(a)** and bowel with signs of hypoperfusion after shock **(b)** upon admission.

The patient received a sodiumbicarbonate- and isotonic saline-bolus. Computed tomography (CT) showed a shock bowel after prolonged resuscitation and profound bilateral pulmonary aspiration, without signs of cerebral edema (Figure 1). The Hallmann oxygenation index was calculated at 10,3 and the patient admitted on the PICU.

Within the following 2 h the patient developed fulminant multi-organ failure. Ventilation had to be intensified to 32 cm H_2_O inspiratory pressure and 18 cm H_2_O PEEP with a resulting tidal volume of 4–6 mL/kg bodyweight. Bronchoscopy revealed no foreign bodies. The patient had general respiratory insufficiency with persistent hypercarbia (pCO_2_ 73 mmHg) and hypoxemia (paO_2_ 37 mmHg) with a reduced peripheral oxygen-saturation (53%). The norepinephrine dosage was increased to 0,25 µg/kg/min and epinephrine to 0,1 µg/kg/min. Echocardiography showed acceptable cardiac function. Laboratory parameters showed elevated levels for creatin-kinase (CK 1,298 U/L), troponin T (819 ng/L) and proBNP (42 pg/mL) and lactate (11 mmol/L). Diuresis was reduced with elevated levels of serum-creatinin (1,17 mg/dL), urea (22 mg/dL) and serum-potassium (6,0 mmol/L) indicating renal failure. Due to impaired coagulation the patient showed increasing nasal and oral bleeding requiring a nasal tamponade insertion.

After performing ROTEM®-Testing we administered tranexamic acid, calciumgluconate, fibrinogen, prothrombin complex concentrate (PPSB) and vitamin K. Neurological status was stable under sedation with midazolam and analgesia with fentanyl. Continuous amplitude-integrated electroencephalography (aEEG) monitoring showed generalized slowing of activity without signs of seizures. Pupils were symmetrically narrow and responsive.

Due to hemodynamic instability, coagulopathy, active systemic inflammatory response syndrome and reduced renal function the patient was reevaluated for ECMO 3 h after admission to the PICU. The Hallmann oxygenation index was now calculated to be 37,3, which in combination with bilateral pulmonary infiltrations on chest-CT-scan indicated severe pARDS according to the revised 2023 criteria (PALICC-2) (8). Neuromaging and bedside continuous aEEG showed no signs of severe cerebral edema as possible contraindication for ECMO.

After fluid resuscitation veno-arterial ECMO was established by bilateral femoral surgical access (19 Fr. venous and 13 Fr. arterial cannula; 4,000 U/min; 2,5 L/min; FiO_2_ 0,6). After stabilization, the patient was transferred to the cardiac surgical ICU.

**Figure 2:**
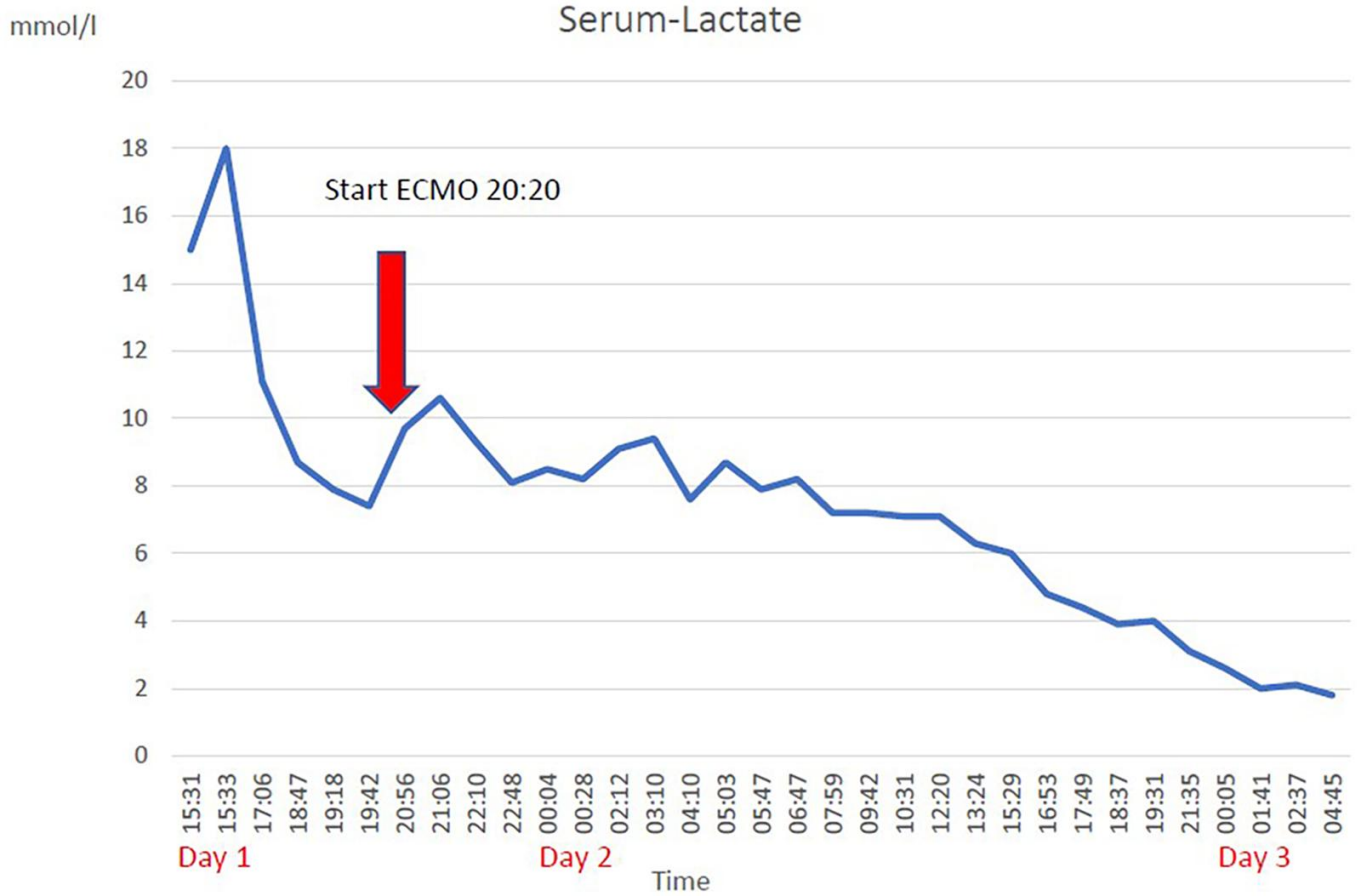
Serum lactate concentration around ECMO administration until normal values.

Coagulation was adjusted to ECMO requirements by continuous administration of heparin to a target PTT of 50-60s. Initial bleeding required administration of packed red blood cells, platelets, fibrinogen and calcium. Ventilation could soon be reduced as well as catecholamine dosages with normalized serum-lactate levels as shown in Figure 2. With recurrent sonographic evaluation of the abdomen and measurement of intraabdominal pressure there were no signs of abdominal compartment syndrome. After 4 days ECMO was discontinued and the patient was transferred back to the PICU. Invasive ventilation was discontinued on day 5 and the patient showed a mild delirium which was managed with clonidine.

Subsequently he was moved to the pediatric neurology department and rehabilitative treatment later on. Upon discharge from the hospital, the patients` neurological status had reached preexisting levels according to the patient and his parents. Three months after the accident an extended neuro-cognitive evaluation [Snijdeŕs-Oomen-nonverbal Intelligence testing 6-40 (SON-R 6-40), Hamburg Writing test 5-10 B (HSP 5-10 B), Child behavior checklist 6-18 R (CBCL 6-18 R), Depression-Inventory for children and adolecents (DIKJ), Inventory for life quality in children (ILK)] showed an IQ-level of 87 with signs of depression and reduced quality of life which were attributed to the preexisting disease.

## Retrospective evaluation of childhood drowning cases

We identified 40 patients suffering from a drowning event between January 1st 2013 and August 31st 2023. All could be included in the case series. Average age was 4,6 years (3 months to 17,5 years) with 34 male and 6 female patients. Three patients died, all of them male, thus overall mortality was 7,5%. Only 4 out of 40 children (10%) had adequate swimming skills. Core epidemiologic and clinical data are summarized in Table 2.

**Table 2:** Demographic and clinical characteristics of the 10-year-cohort.

Parameter	Overall (*n* = 40)	Survivors (*n* = 37)	Non-survivors (*n* = 3)
Age in years (average)	4,55 (0,25–17,5)	4,46 (0,25–17,5)	5,67 (1,67–10,17)
Sex (male/female)	34/6	31/6	3/0
Water temperature >20 °C/ < 20 °C	32/8	30/7	2/1
Median submersion time in minutes	3,7 (0,3–15)	3 (0,3–15)	10 (5–15)
Cardiopulmonary resuscitation (no of cases)	30/40	–	–
CPR duration < 5 minutes (no of cases)	23/30	23	0
CPR duration >20 minutes (no of cases)	7/30	4	3
Mean body temperature ( °C) at EMS site	34,3 (26,2–36,8)	35,3 (26,2–36,8)	31 (30,2–32,1)
pH at ED (average)	7,24 (6,63–7,46)	7,28 (6,67–7,46)	6,87 (6,63–7,38)
Average serum lactate concentration (mmol/L) at ED	5 (0,8–24)	3,9 (0,8–15)	16,2 (1,6–24)
Median PIM-3-score at PICU-arrival	–	8,1 (0,3–93,4)	94,7 (32,6–99,4)

### Preclinical setting

**Figure 3:**
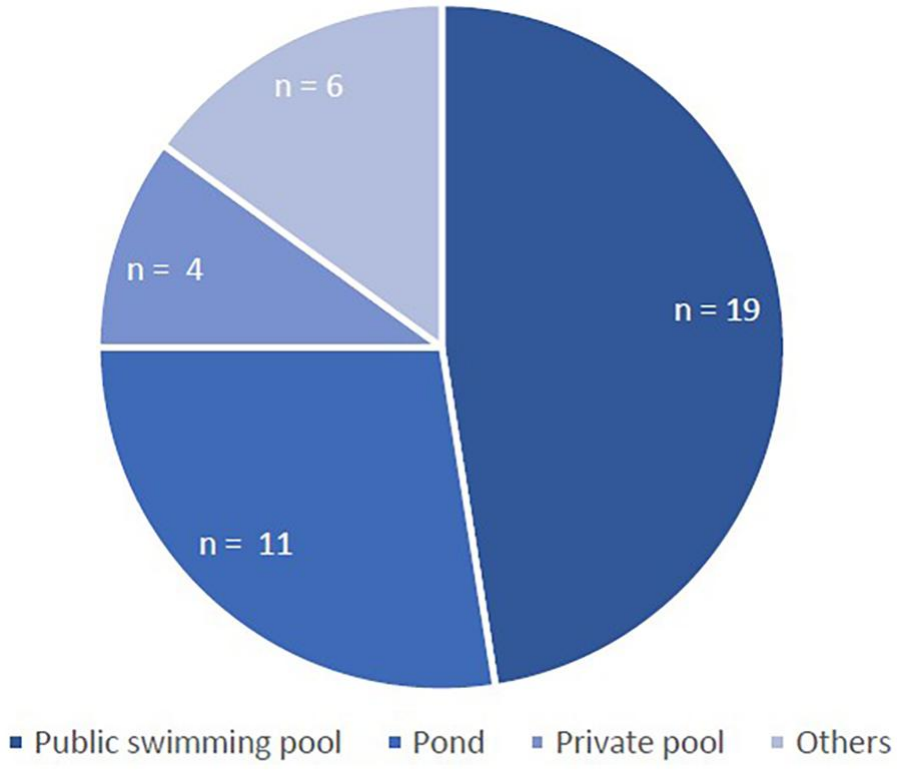
Locations of drowning.

Drowning happened under different circumstances. 19 patients drowned in a public swimming pool, 11 in a pond, 4 in a private pool and another 6 in miscellanous locations, e.g., a canal (Figure 3).

Drowning happened mostly in sweet-water (*n* = 36), but as the Oldenburg area is located near the german north sea coast, 4 patients experienced saltwater-drowning. This had no effect on clinical course or mortality. Two of 40 patients had additional minor injuries from the accident.

Water temperature was estimated as reported by the EMS team. Of 32 patients drowning in water warmer than 20 °C 2 patients died, whereas 1 of 8 patients retrieved from water under 20 °C died [Fisher Exact Test, *p* > 0,05, Odds ratio (OR) 2,29]. Submersion time was estimated by anamnestic information retrieved on site. Median reported submersion time was 3,7 min (range: 0,3–15 min). Surviving patients had median submersion times of 3 min, whereas median submersion times for patients dying in the PICU were 10 min.

Cardiopulmonary resuscitation (CPR) was performed in 30/40 (75%) cases and was often primarily initiated by lay bystanders. In 22/40 patients the preclinical cardiac rhythm was reported as asystole or PEA. Duration of CPR was under 5 min in 23/30 patients and none of these children died. For patients with CPR duration longer than 20 min (7/30 cases), 3 patients died later on (Fisher Exact Test, *p* = 0,22, OR 21,93).

Median body temperature at the site of EMS treatment was 34,3 °C (Range 26,2–36,8 °C; reported in 34 of 40 patients). Survivors had median temperatures of 35,3 °C, whereas children who deceased in-hospital had a median temperature of 31 °C in the preclinical setting. Patients who were breathing spontaneously on site before transport to the ED had a mean GCS score of 13,1.

### Data from the ED

All patients were received at the ED by an interdisciplinary team of PICU physicians, anesthesiologists, radiologists and emergency physicians. After initial stabilization and imaging studies the patients were referred to the PICU. Median temperature in the ED was 35,8 °C (Range 25,4–36,8 °C; reported in 39/40 cases). None of the patients had relevant serum-potassium elevations (average 3,9 mmol/L; range 3,1–6,3 mmol/L). There was a trend of lower pH in the non-survivors (pH 6,87 vs. 7.28 for survivors) and elevated serum-lactate levels in the non-survivors (16,2 vs. 3,9 mmol/L for survivors). On arrival at the ED, 7/30 post-CPR patients required catecholamine-support and 9/30 were mechanically ventilated. The average GCS score of patients breathing spontaneously upon admission was 14,8.

### PICU data

All 40 patients were referred to the PICU from the ED. Upon arrival, 7/40 (17,5%) patients required catecholamine-support and 11/40 (27,5%) were mechanically ventilated. Non-survivors were all catecholamine-dependent and had lower body temperatures (median temperature 31 °C vs. 35,3 °C in survivors). Patients who needed prolonged CPR longer than 20 min after drowning were likely to be catecholamine-dependent on PICU-arrival (6/7, 85,7%). Average duration of ventilation was 88,3 h. Patients breathing spontaneously upon arrival had an average GCS of 13,1. Average length of stay was 4,5 days (median: 2 days).

### Outcome data

Overall mortality was 3/40 (7,5%) with an average patient age of 5,67 years for non-survivors. Post-CPR mortality was 10% (3/30). There was no significant difference for mortality after drowning in sweet- or salt-water (Fisher Exact Test, *p* = 0,316, OR = 0,2055). All 3 patients died during their stay on the PICU within 48 h due to untreatable hypoxic brain edema on CT- or Magnetic-Resonance-Imaging (MRI). Therefore, ECMO was not considered in these patients.

When scoring patients for Pediatric Index of Mortality (PIM-3) upon PICU-arrival survivors (*n* = 37) had a significantly lower median score of 8 compared to non-survivors (*n* = 3; median score 94; Fisher Exact-Test, *p* < 0,001, OR = 0,01).

Neurologic status at PICU discharge was calculated with FSS (Functional status scale) (7) and compared to FSS prior to the drowning accident. Median FSS pre and post-PICU treatment was 6. In individual patients, 4/36 patients (11,1%) showed differences in FSS-Scores > 2 (median delta FSS=11).

## Discussion

Drowning is still one of the leading causes of morbiditiy and mortality in pediatric accidents world wide. The incidence is highest in small children and teenagers without adequate swimming skills (1, 9, 10). Established risk factors for non-survival and poor neurologic outcome are submersion time, water temperature and prolonged resuscitation (>30 min) (9–13). Additionally there are influencing conditions like acidosis, elevated serum lactate levels and the development of ARDS which may affect prognosis (3, 14, 15). Persistent hypothermia upon arrival at the ED is considered to be unfavorable for survival. However, drowning in ice cold water may be neuroprotective due to the immediate reduction of neurometabolic activity (16). Preclinical management focuses on reversing hypoxia and re-establishing adequate circulation without specific differences between pediatric and adult cases (17, 18). However, despite these well-established risk factors, giving an adequate prediction regarding outcome during primary care situations remains difficult (15).

Based on these findings we could demonstrate that in our cohort of 40 patients collected over a timespan of 10 years most of these risk factors hold true. Thus, the characteristics of pediatric drowning patients in our area should be comparable to established populations in other studies.

Compared to our group of patients, the case we report represents a significant outlier. The patient required prolonged CPR over 95 min without severe hypothermia and developed a severe ARDS within the first hours in the PICU requiring ECMO. There was concomitant lactic acidosis and hypoxemia. Therefore, he initially had a poor prognosis with respect to survival and neurologic outcome. Suprisingly, he recovered rapidly with ECMO and was eventually discharged in a good neurologic condition.

There are few publications reporting comparable cases. Livshits et al. reported a 6 year old boy after CPR for 57 min of resuscitation with a submersion time between 10 and 15 min. He developed ARDS without the need for ECMO and had a good neurologic status on follow-up 2 years later (19). Eich et al. reported a 3 year old girl with resuscitation time of 140 min and severe hypothermia. She needed ECMO for rewarming and 4 consecutive days of ECMO for overcoming a severe ARDS. She initially remained in a vegetative state and was transferred for rehabilitation. After 2,5 years she had improved markedly and showed minor neurologic restrictions (20). A case of a 66 year old adult with favorable neurologic outcome after CPR for 20 min and 12 days of ECMO for ARDS was published by Cho et al. (21). When analyzing these cases there seem to be no common characteristics that explain the significantly different clinical course in comparison to the majority of cases with poor prognostic risk factors.

The development of severe respiratory failure after drowning is a complication leading to higher morbidity and mortality in adults and children of all ages (4). Treating lung injury and reversal of hypoxemia are the main goals, for which multiple ECMO-applications from E-CPR at the ED to vv- or va-ECMO for secondary ARDS are established options (22). ELSO registry data show survival rates after ECMO ranging from 71,4% for patients without cardiac arrest to 23,4% in children receiving E-CPR after drowning (23). Besides the above mentioned case reports there are no larger studies reporting the incidence of ARDS and the need for ECMO in pediatric drowning cases.

Whereas for adults the justification for ECMO or E-CPR is a given due to the potentially reversible nature of the symptoms (24), there is no specific guideline for pediatric drowning with or without ARDS due to the lack of evidence (25). Pediatric ECMO seems to have a higher survival rate than in adult patients (26). However, there is a substantial risk for severe neurologic complications as cerebral bleeding or ischemia in 10% of the treated patients (27), thus reducing the chance of a favorable outcome. As was the case in our patient, a common indication for ECMO is an oxygenation index >16 and lack of clinical improvement under conventional mechanical ventilation. An oxygenation index >40 is associated with a higher mortality in pediatric ECMO patients. Nevertheless, as Polito et al. stated in their analysis of recent ELSO data, the "optimal timing for ECMO deployment is not known“ (28).

As decision making regarding ECMO in pediatric ARDS after drowning is not supported by evidence, there are some hints regarding long-term outcome. Pediatric patients seem to have a better 30-day-survival than adults, but the risk for neurologic impairment after drowning is similar (29). When CPR is necessary post-drowning there is a significantly higher risk of multi-organ failure and neurologic impairment (2). Normothermia after resuscitation showed the same outcome as therapeutic hypothermia, without the hypothermia-associated side effects (30). Normothermia is therefore recommended in post-resuscitation care. Currently, there are no reliable prognostic scales that could distinguish between children with high or low risk for longterm neurologic problems (15).

Our study is limited due to low patient-count, which allows only for a descriptive statistical analysis, especially when looking at mortality rates. However, due to the geographical characteristics of north-western Germany we report a comparable percentage of cases given the amount of inhabitants such as Nitta et al. did for the Osaka region (29). With calculating PIM-3-Scores and FSS-Values retrospectively from patient records there is a substantial risk of unrecognized bias. We chose PIM-3 for its known resilience to missing data and could demonstrate that it discriminates well between patients with high and low mortality risk upon retrospective analysis. PIM-3 does not allow for correct estimation of the clinical course in individual cases. As expected, study size did not allow for thorough analysis of neurologic outcome by FSS-scale.

## Conclusion

Drowning is one of the leading causes of morbidity and mortality in pediatric accidents. Although there are established prognostic parameters to evaluate risk for poor outcome, there are few cases with good long-term neurologic outcome against all odds. Our case, with prolonged preclinical resuscitation and secondary severe ARDS requiring ECMO, represents such an unusual course when compared to the local 10-year experience. Currently, there is no single parameter that aids in distinguishing between these regular and special cases. Therefore, deciding which treatment option is fitting for each individual patient depends on personal experience and individual patient parameters. Further studies on these topics are warranted.

## Data Availability

The original contributions presented in the study are included in the article/Supplementary Material, further inquiries can be directed to the corresponding author.
